# Dual vs. mono antiplatelet therapy for acute ischemic stroke or transient ischemic attack with evidence of large artery atherosclerosis

**DOI:** 10.3389/fneur.2022.923142

**Published:** 2022-09-12

**Authors:** Chun-Jen Lin, Tzu-Yun Tseng, Jeffrey L. Saver

**Affiliations:** ^1^Neurological Institute, Taipei Veterans General Hospital, Taipei, Taiwan; ^2^School of Medicine, National Yang Ming Chiao Tung University, Taipei, Taiwan; ^3^Department of Internal Medicine, Far Eastern Memorial Hospital, New Taipei, Taiwan; ^4^Comprehensive Stroke Center and Department of Neurology, David Geffen School of Medicine, University of California, Los Angeles, Los Angeles, CA, United States

**Keywords:** dual antiplatelet therapy, large artery atherosclerosis (LAA), ischemic stroke, meta-analysis, clopidogrel, cilostazol, ticagrelor

## Abstract

**Background and purpose:**

Current pieces of evidence support the short-term use of dual antiplatelet (DAPT) in minor ischemic stroke or transient ischemic attack (TIA) based on the studies performed in patients with a broad range of non-cardioembolic stroke mechanisms. However, the efficacy and safety of DAPT use in ischemic stroke patients with large artery atherosclerosis (LAA) are still uncertain. We undertook a systemic search and formal meta-analysis to compare DAPT vs. mono-antiplatelet therapy (MAPT) in patients with etiology specifically presumed to be symptomatic LAA.

**Methods:**

We conducted a systemic online search for completed randomized controlled trials that (1) compared DAPT vs. MAPT in patients with acute ischemic stroke or TIA, and (2) were confined to or had available subgroup data regarding population with symptomatic extra- or intracranial artery stenosis. Study-level meta-analysis was performed for outcomes, including ischemic stroke (IS) recurrence, intracranial hemorrhage (ICH), and major bleeding with the Mantel-Haenszel method and random effect models, and was described as risk difference (RD) and 95% CI.

**Results:**

A total of 10 trials including 5,004 patients were pooled. Comparing to MAPT, DAPT significantly reduced IS recurrence (5.99 vs. 9.55%, RD: −3%, 95% CI: −5**–**0%). Across all agents, out of 100 treated patients, 3 fewer had a recurrent ischemic stroke with DAPT. The safety endpoints including ICH (0.28 vs. 0.32%, RD: 0%, 95% CI: −0**–**0%) and major bleeding (0.73 vs. 0.51%, RD: 0%, 95% CI: −0**–**0%) did not differ significantly.

**Conclusion:**

In patients with symptomatic large artery extracranial or intracranial atherosclerosis, DAPT was superior to MAPT in preventing IS recurrence without increasing bleeding risks. The optimal DAPT regimens and duration of treatment in this population need to be clarified in further studies.

## Introduction

Large artery atherosclerosis (LAA) accounts for 15–40% of ischemic strokes (1, 2). The best strategy for antiplatelet therapy to prevent recurrent stroke in patients with symptomatic LAA is uncertain. Compared with mono-antiplatelet therapy (MAPT), intensive, double antiplatelet agent therapy (dual antiplatelet therapy, DAPT) is physiologically expected to better avert recurrent plaque-site thrombosis and recurrent ischemic stroke but also to increase the risk of cerebral and systemic bleeding.

Several clinical trials and meta-analyses have compared MAPT vs. DAPT for the prevention of recurrent ischemic stroke, and shown heterogeneous results in efficacy and increased bleeding risks (3–6). However, their topline results have typically focused upon a broad overall trial population consisting of both patients with small vessel disease, as well as patients with LAA. This mixed population may mask the distinctive effects of intensive antiplatelet therapy in patients with LAA. LAA patients have greater plaque surface area and so potentially greater proneness to platelet activation and aggregation, possibly increasing the efficacy of DAPT. Further, small vessel disease patients often have more extensive cerebral microbleeds and bleeding prone to microangiopathy, potentially magnifying the risk of DAPT (3, 7).

Given these considerations, it is desirable to pool available data specifically in symptomatic LAA patients from completed randomized trials of MAPT vs. DAPT to delineate the specific benefits and risks in this patient population. Accordingly, we undertook a systematic meta-analysis of MAPT vs. DAPT in transient ischemic attack (TIA) or ischemic stroke patients with evidence of cervico-cerebral artery atherosclerosis.

## Materials and methods

The meta-analysis was performed according to the recommendations of the Preferred Reporting Items for Systematic Reviews and Meta-Analyses (PRISMA) statement (8). We searched PubMed, EMBASE, Web of Science, Cochrane Collaboration Central Register of Controlled Clinical Trials, and the clinical trial registry maintained at https://www.clinicaltrials.gov from January 2004 to December 2021 using the search strategy of “dual antiplatelet” or “aspirin plus clopidogrel” or “aspirin plus cilostazol” or “aspirin plus ticlopidine” or “aspirin plus dipyridamole” or “aspirin plus ticagrelor” AND “compare” or “vs.” AND “aspirin” AND “large artery atherosclerosis” or “carotid artery stenosis” or “intracranial artery stenosis” or “intracranial atherosclerotic diseases” AND “stroke” or “transient ischemic attack.” We restricted the results to studies performed on human beings and those published in English. Additional studies were identified in the texts and reference lists in retrieved articles.

Criteria for study inclusion in the meta-analyses were: (1) randomized controlled trial comparing recurrent vascular event rates with DAPT vs. MAPT; and (2) separately reported data for patients with stroke or TIA and with evidence of cervico-cerebral artery atherosclerosis. Studies with patients receiving either anticoagulants or thrombolysis would be excluded. All data were extracted independently by 2 investigators (C-J.L. and J.L.S.), with discrepancies resolved by consensus discussion. Study quality was assessed with the Cochrane Risk of Bias 2 (RoB 2) tool.

We performed the meta-analysis with Review Manager (RevMan), Version 5.4.1 (Cochrane Collaboration, 2020). The efficacy endpoint was a recurrent ischemic stroke. The safety endpoints were intracranial hemorrhage (ICH) and major bleeding according to the definition of the original studies. We compared the outcomes between DAPT vs. MAPT by calculating the risk difference (RD) and 95% confident interval (CI) using the Mantel-Haenszel method and a random-effects model. Publication bias was estimated visually by funnel plots displaying standard errors as the measure of sample size and RD as the measure of treatment effect. Statistical significance was generally set at *p* < 0.05. For heterogeneity testing, *p* = 0.05–0.1 was considered as evidence of possible heterogeneity.

## Results

**Figure 1:**
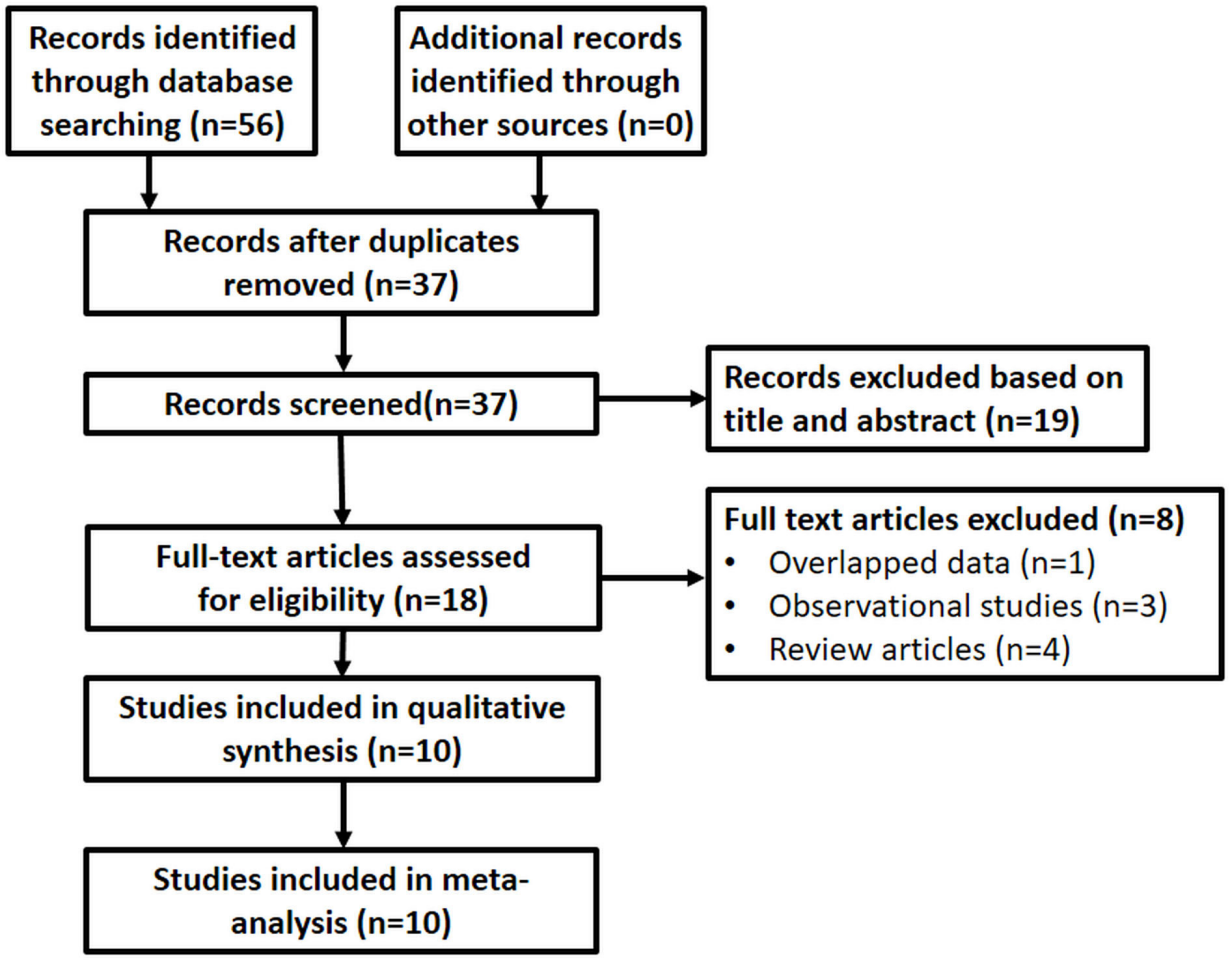
Flowchart of article selection. The literature search strategy identified 53 records initially. After the detailed screening, ten trials, enrolling 5,004 individuals, fulfilled all selection criteria and were included in the meta-analysis.

The literature search strategy identified 56 records initially. After the detailed screening, ten trials, enrolling 5,004 individuals, fulfilled all selection criteria and were included in the meta-analysis (Figure 1).

Table 1 shows the key characteristics of included studies. All studies were designed as randomized controlled trials (9–14), including seven studies solely enrolling patients with LAA and three studies reporting LAA subgroup analyses of the original randomized controlled trials (15, 16). Among the trials, six compared aspirin plus clopidogrel vs. aspirin alone, three compared cilostazol plus aspirin vs. aspirin alone, and one compared ticagrelor plus aspirin vs. aspirin alone. Steno-occlusive sites included: any extracranial internal carotid artery (E-ICA), anterior circulation intracranial atherosclerotic disease (ICAD), or posterior ICAD in five trials; solely ICAD in four trials, and solely E-ICA in one trial. Qualifying events were TIA or mild ischemic stroke in three trials; TIA or mild-major ischemic stroke in two trials; and mild-major ischemic stroke without TIA in five trials. Across all studies, the permitted onset to randomization time ranged from within 2 days to within 3 months, and among trials reporting actual time to randomization, the great preponderance of enrollments within the first 24–48 h. The duration of DAPT treatment ranged from 7 to 90 days for aspirin plus clopidogrel or ticagrelor and was 6 months to more than 1 year for aspirin plus cilostazol.

**Table 1:** Design and population characteristics of included trials.

**Study**	**CARESS**	**TOSS**	**CLAIR**	**Yi et al**.	**CATHARSIS**	**CHANCE**	**COMPRESS**	**Zuo et al**.	**THALES**	**CSPS.COM**
Year	2005	2005	2010	2014	2015	2015	2016	2017	2020	2021
No. of Participants	107	135	98	570	163	481	352	200	2,351	547
Age	66.4 vs. 62.8	62.2 vs. 62.5	59.2 vs. 56.4	69.2 vs. 70.1	68.3 vs. 68.3	65.9 vs. 65.8	68 vs. 67	61.6 vs. 62.3	67.1 vs. 67.6	70 vs. 70
Male	68.6 vs. 69.6%	61.2 vs. 60.3%	78 vs. 77%	54.9 vs. 54.9%	77.1 vs. 53.8%	64.5 vs. 60.8%	65.5 vs. 61.7%	60.6 vs. 59.7%	67.5 vs. 68.1%	63.3 vs. 71.0%
Race-Ethnicity	Europeans	Asians	Asians	Asians	Asians	Asians	Asians	Asians	Global	Asians
Definition of LAA	E-ICA stenosis 50–99/100%	M1 or BA stenosis	E-ICA, I-ICA, or M1 stenosis 50–99/100%	LAA per TOAST	I-ICA, M1, BA 50–99/100%	I-ICA, M1/2, I-VA, BA 50–99/100%	E-ICA stenosis 30–99/100% or ICAD	E-ICA, I-ICA, MCA, I-VA, BA, PCA 50–99/100%	Ipsilateral vessel 30–99/100%	I-ICA, MCA, ACA, PCA 50–99/100%
TIA as Qualifying Event	62.7 vs. 60.7%	0%	Proportion NR	0%	0%	23.8 vs. 22.8%	0%	50 vs. 47%	13.9 vs. 14.4%	0%
Proportion of ICAD among LAA	0%	100%	98 vs. 92%	NR	100%	100%	70.1 vs. 75.4%	NR	45.4 vs. 45.9%	100%
NIHSS allowed for randomization	≤ 22	≤ 15	≤ 8	≤ 12	Any	≤ 3	Any	Any	≤ 5	Any
Mean/Median NIHSS	NR	NR	1 vs. 1	11.2 vs. 11.5	NR	NR	3 vs. 3	NR	56% 1–3	NR
Time Window for Randomization	≤ 3 months	≤ 2 weeks	≤ 7 days	≤ 2 days	2 weeks to 6 months	≤ 24 h	≤ 48 h	≤ 7 days	≤ 24 h	8**–**180 days
Mean Time Onset to Randomization	77.1% within 1 month	NR	2.5 vs. 3.2 d	26 vs. 24 h	NR	10.5 vs. 13h	35.2 vs. 33.5h	NR	NR	NR
Medications	ASA + CLOP vs. ASA	ASA + CILO vs. ASA	ASA + CLOP vs. ASA	ASA + CLOP vs. ASA	ASA + CILO vs. ASA	ASA + CLOP vs. ASA	ASA + CLOP vs. ASA	ASA + CLOP vs. ASA	ASA + TICA vs. ASA	ASA + CILO vs. ASA
Duration of DAPT treatment	7 days	6 months	7 days	30 days	2 years	3 weeks	30 days	90 days	30 days	1.4 years
Duration of Follow-up	7 days	6 months	7 days	30 days	2 years	90 days	30 days	90 days	30 days	1.4 years

ASA indicates aspirin; BA, basilar artery; CLOP, clopidogrel; CILO, cilostazol; E-ICA, extracranial internal carotid artery; I-ICA, intracranial internal carotid artery; ICAD, intracranial atherosclerotic disease; I-VA, intracranial vertebral artery; M1/M2, M1/M2 segment of the middle cerebral artery; MCA, middle cerebral artery; NIHSS, National Institutes of Health Stroke Scale; NR, not reported; PCA, posterior cerebral artery; RCT, randomized controlled trial; TIA, transient ischemic attack; TICA, ticagrelor; TOAST, Trial of ORG 10172 in the Acute Stroke Treatment classification system.

The quality assessment of the retrieved trials on the RoB 2 is shown in Supplementary Figure 1. Six out of ten trials were double-blind in study design and had a low risk of bias in all five domains. Four trials using open-label design showed a moderate risk of bias in the domain of deviations from intended interventions in all trials and the domain of measurement of the outcome in one trial, with a low risk of bias in the other three domains. The funnel plots showed no major distribution deviation of publications (Supplementary Figures 2–4).

**Figure 2:**
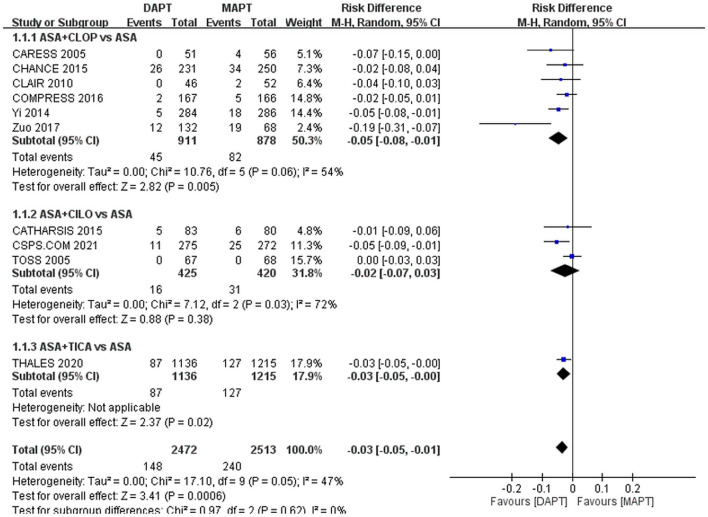
Forest plot for outcome of ischemic stroke. Compared to MAPT, DAPT significantly reduced ischemic stroke recurrence in patients with symptomatic large artery atherosclerosis. ASA, aspirin; CILO, cilostazol; CLOP, clopidogrel; DAPT, dual antiplatelet therapy; MAPT, mono antiplatelet therapy, M-H, Mantel-Haenszel method; TICA, ticagrelor.

Table 2 shows the rates of ischemic stroke recurrence, ICH, and major bleeding in each of the included studies. The pooled analysis showed that overall DAPT compared with MAPT was associated with a significant reduction in recurrent ischemic stroke, 5.99 vs. 9.55%, RD: −3%, 95% CI: −5**–**0%, *p* = 0.002 (Figure 2). There was no significant heterogeneity by agent subgroups (*p* = 0.62, I^2^ = 0%).

**Table 2:** Outcomes of included studies.

**Study**	**CARESS**	**TOSS**	**CLAIR**	**Yi et al**.	**CATHARSIS**	**CHANCE**	**COMPRESS**	**Zuo et al**.	**THALES**	**CSPS.** **COM**
Recurrent IS - events	0/51 vs. 4/56	0/67 vs. 0/68	0/46 vs. 2/52	5/284 vs. 18/286	5/83 vs. 6/80	26/231 vs. 34/250	2/167 vs. 5/166	12/132 vs. 19/68	87/1,136 vs. 127/1,215	11/275 vs. 25/272
Recurrent IS - rate (% per week)	0 vs. 7.1%	0 vs. 0%	0 vs. 3.9%	0.4 vs. 1.5%	0.1 vs. 0.1%	0.9 vs. 1.1%	0.3 vs. 0.7%	0.7 vs. 2.2%	1.9 vs. 2.6%	0.1 vs. 0.1%
ICH - events	0/51 vs. 0/56	0/67 vs. 0/68	0/46 vs. 0/52	1/284 vs. 1/286	0/83 vs. 2/80	0/231 vs. 0/250	1/167 vs. 0/166	0/132 vs. 0/68	4/1,136 vs. 3/1,215	1/275 vs. 2/272
ICH - rate (% per week)	0 vs. 0%	0 vs. 0%	0 vs. 0%	0.1 vs. 0.1%	0 vs. 0%	0 vs. 0%	0.14 vs. 0%	0 vs. 0%	0.08 vs. 0.06%	0 vs. 0%
Major bleeding - events	0/51 vs. 0/56	0/67 vs. 0/68	0/46 vs. 0/52	1/284 vs. 1/286	4/83 vs. 3/80	0/231 vs. 1/250	7/174 vs. 2/178	0/132 vs. 0/68	6/1,136 vs. 3/1,215	2/274 vs. 3/272
Major bleeding - rate (% per week)	0 vs. 0%	0 vs. 0%	0 vs. 0%	0.1 vs. 0.1%	0.1 vs. 0%	0 vs. 0.03%	0.94 vs. 0.26%	0 vs. 0%	0.13 vs. 0.06%	0 vs. 0%

ICH, indicates intracranial hemorrhage; IS, ischemic stroke.

**Figure 3:**
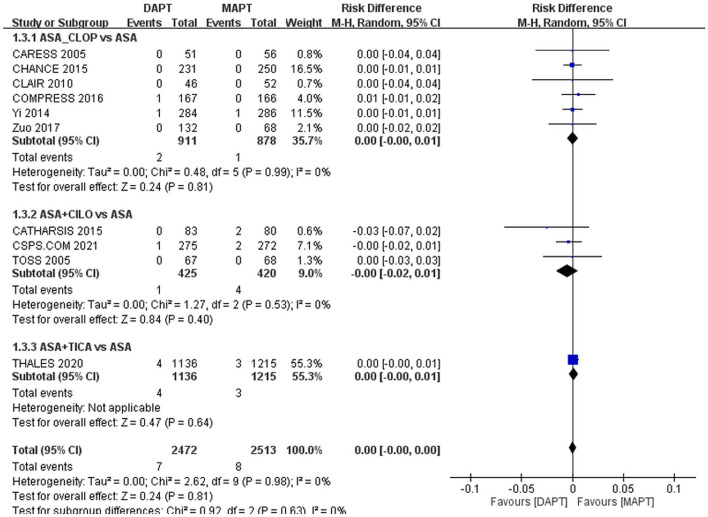
Forest plot for outcome of intracranial hemorrhage. The risk of intracranial hemorrhage was not significantly different between MAPT and DAPT in patients with symptomatic large artery atherosclerosis. ASA, aspirin; CILO, cilostazol; CLOP, clopidogrel; DAPT, dual antiplatelet therapy; MAPT, mono antiplatelet therapy, M-H, Mantel-Haenszel method; TICA, ticagrelor.

**Figure 4:**
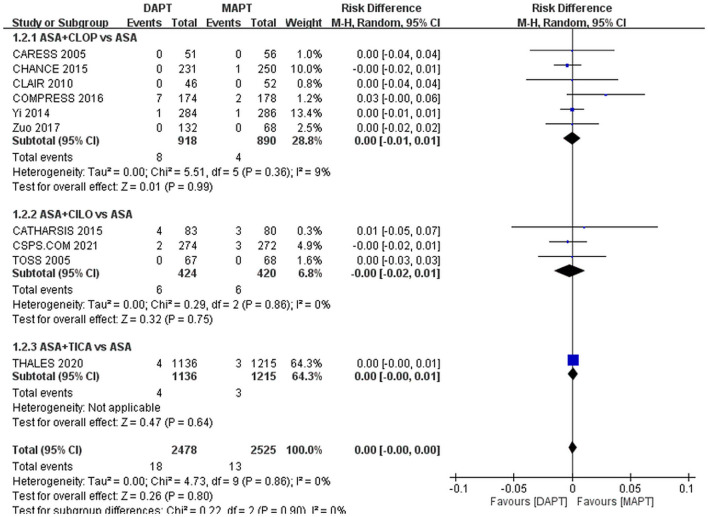
Forest plot for outcome of major bleeding. The risk of major bleeding wa not significantly different between MAPT and DAPT in patients with symptomatic large artery atherosclerosis. ASA, aspirin; CILO, cilostazol; CLOP, clopidogrel; DAPT, dual antiplatelet therapy; MAPT, mono antiplatelet therapy, M-H, Mantel-Haenszel method; TICA, ticagrelor.

Safety endpoint rates did not differ significantly between the DAPT and MAPT groups: ICH, 0.28 vs. 0.32%, RD: 0%, 95% CI: −0**–**0% (Figure 3); major bleeding,0.73 vs. 0.51%, RD: 0%, 95% CI: −0**–**0% (Figure 4).

## Discussion

The present systemic review and meta-analysis compared the efficacy and safety of DAPT vs. MAPT in patients with ischemic stroke or TIA with evidence of large artery atherosclerosis. The pooled results showed that compared to MAPT, DAPT significantly reduced ischemic stroke recurrence, without increasing the risk of ICH or major bleeding. Across all agents, out of 100 treated patients, 3 fewer had a recurrent ischemic stroke with DAPT.

This study is consonant with and extends prior investigations. Several trials and meta-analyses have demonstrated an advantage of short courses of DAPT over MAPT early after index event among patients with a broad range of non-cardioembolic stroke mechanisms, including large artery atherosclerosis, small vessel disease, and cryptogenic stroke (4, 6, 17). However, to our knowledge, this is the first meta-analysis elucidating the role of DAPT specifically in patients with symptomatic LAA. The findings of the benefit of early DAPT for symptomatic LAA in the cerebral circulation accords with the established benefit of early DAPT in patients with symptomatic LAA in the cardiac circulation (18, 19). Ischemic strokes due to LAA have been reported to have higher early recurrence rates than other stroke mechanisms (20, 21), making patients with LAA especially likely to benefit from an initial course of DAPT.

Distinctive aspects of the study populations and study regimens in this meta-analysis should be borne in mind when applying findings to clinical practice. First, the treatment regimen was administered in the preponderance of patients for an abbreviated period after index TIA or ischemic stroke, including 21 days in the Clopidogrel in High-Risk Patients with Acute Nondisabling Cerebrovascular Events (CHANCE) trial and 30 days in the Acute Stroke or Transient Ischemic Attack Treated with Ticagrelor and ASA for Prevention of Stroke and Death (THALES) trial. Compared with the heart, the brain is more vulnerable to hemorrhage on DAPT therapy, and with long-term DAPT therapy increased hemorrhagic stroke rates offset reduced recurrent ischemic stroke rates. But the risk of recurrent ischemic stroke is especially high in the first weeks after an index LAA ischemic event, so in this time period, the benefit of DAPT outweighs the risk (21–23). The time of the start of antithrombotic therapy was within the first 24 h of the index event in two of the analyzed trials but the remainder had a longer enrollment window. The benefit magnitude in trials with later enrollment was similar to those confined to the first 24 h. Accordingly, the results of the current study suggest that if evidence of LAA is identified, DAPT may still be considered even if treatment would be initiated beyond the first 24 h after the index cerebral ischemic event.

The preponderance of patients enrolled in the analyzed trials had a transient ischemic attack or minor ischemic stroke as their presenting event, with few evidencing major ischemic stroke. The risks of hemorrhagic transformation on antithrombotic therapy increase with infarct volume, so risk-benefit ratios may differ between minor and major cerebral ischemic events (24). Accordingly, the current findings provide support for the use of early DAPT in LAA patients with TIA or minor ischemic stroke. Among four included trials reporting information about the mean/median NIHSS of enrolled patients (CLAIR, (12), COMPRESS, THALES), only one trial (12) randomized patients with moderate stroke (NIHSS 11.2 and 11.5 in DAPT and MAPT group, respectively). The potential advantages of early DAPT vs. MAPT in LAA patients with moderate to major ischemic stroke require clarification in further randomized clinical trials.

Though there was no significant agent heterogeneity in the current meta-analysis, cilostazol added to aspirin did not confer the advantages of clopidogrel or ticagrelor added to aspirin. However, the three trials comparing cilostazol plus aspirin vs. aspirin alone have two distinct features compared to the trials of clopidogrel or ticagrelor: (1) having a longer duration of DAPT, (2) enrolling ICAD subjects only. While previous studies indicated that long-term DAPT with clopidogrel added to aspirin provided limited benefit but increased bleeding risk (5), the current study showed that the safety of long-term DAPT with cilostazol added to aspirin was comparable to aspirin alone in patients with symptoms of ICAD.

In addition to DAPT, other combinations of antithrombotic agents including direct oral anticoagulants are being tested for stroke prevention in individuals of high ischemic risk. An ongoing phase 3 prospective multicenter double-blinded randomized controlled trial, a comparison of anti-coagulation vs. antiplatelet therapies for intracranial vascular atherosclerosis (CAPTIVA), was designed to compare low-dose rivaroxaban plus aspirin to the best DAPT (clopidogrel + aspirin or ticagrelor + aspirin according to the CYP2C19 genotype testing) in symptomatic 70–99% intracranial stenosis (ICAD) for preventing one-year recurrent ischemic stroke. The results may clarify the effectiveness and safety of an alternative therapeutic option if a P2Y12 inhibitor is not tolerated or for a CYP2C19 LOF allele carrier (25).

There are limitations to this study. First, the definition of LAA was variable based on different criteria. According to the original TOAST classification, LAA was defined as the stenosis of the attributed vessel being 50% or more (26). However, several new classification systems consider not only the degree of stenosis, but also the presence of plaque ulceration, thrombus, or the involvement of penetrating arteries (2, 27). Several recent studies have adopted 30% or greater stenosis to define LAA, or even no limitation for stenotic degree (14, 16, 28). The current meta-analysis included trials with different criteria to define the presence of atherosclerosis. Second, two of the analyzed studies (CARESS and CLAIR) were designed primarily to evaluate a short-term, surrogate outcome (transcranial Doppler embolic signals at day 7), instead of vascular events accrued over a longer time period. Their short follow-up duration may have resulted in underestimates of differential treatment effects. To mitigate this issue, we performed an analysis of the rate of events per week across all studies for comparison. Third, the majority of patients in the included studies were Asian. Especially, the significant benefit of aspirin plus clopidogrel was mainly driven by two Chinese studies (12, 13). The generalizability of the result of this study to non-Asians needs to be confirmed in further investigations.

## Conclusion

In this meta-analysis, we demonstrated that, compared to MAPT with aspirin alone, DAPT early after index cerebral ischemic events in patients with symptomatic LAA provided better ischemic stroke prevention, without increasing ICH or major bleeding risk. The number needed to treat to prevent 1 recurrent stroke was 33. Further studies to clarify the optimal DAPT regimens and duration of treatment in this population are warranted.

## Data availability statement

The original contributions presented in the study are included in the article/Supplementary material, further inquiries can be directed to the corresponding author.

## Author contributions

C-JL: data acquisition, data analysis, and manuscript drafting and revision. T-YT: data analysis, figure making, and manuscript writing. JS: conception and design of the study and revision of the submitted version. All authors contributed to the article and approved the submitted version.

## Funding

This work was funded by the Taiwan Ministry of Science and Technology (MOST 110-2314-B-075-043) and the Taipei Veterans General Hospital (V110B-026).

## Conflict of interest

Author JS is an employee of the University of California. The University of California has patent rights in retrieval devices for stroke. He has served as an unpaid site investigator in multicenter trials sponsored by Medtronic, Stryker, and Neuravia, for which the UC Regents received payments on the basis of clinical trial contracts for the number of subjects enrolled; has received funding for services as a scientific consultant regarding trial design and conduct to Medtronic, Stryker, Neuravi/Cerenovus and Boehringer Ingelheim (prevention only), and stock options for services as a scientific consultant regarding trial design and conduct to Rapid Medical. The remaining authors declare that the research was conducted in the absence of any commercial or financial relationships that could be construed as a potential conflict of interest.

## Publisher's note

All claims expressed in this article are solely those of the authors and do not necessarily represent those of their affiliated organizations, or those of the publisher, the editors and the reviewers. Any product that may be evaluated in this article, or claim that may be made by its manufacturer, is not guaranteed or endorsed by the publisher.
