# Supplementary material for: Dual vs. mono antiplatelet therapy for acute ischemic stroke or transient ischemic attack with evidence of large artery atherosclerosis
Source: Front Neurol. 2022 Sep 12;13:923142. doi: 10.3389/fneur.2022.923142 (PMC9510375; doi:10.3389/fneur.2022.923142)

**Supplementary Materials**

**efigure legends:**

eFigure I. Study quality was assessed with the Cochrane Risk of Bias 2 (RoB 2) tool. Six out of ten trials were double-blind in study design and had low risk of bias in all the five domains. Four trials using open-label design showed moderate risk of bias in the domain of deviations from intended interventions in all trials and in the domain of measurement of the outcome in one trial, with low risk of bias in other three domains.


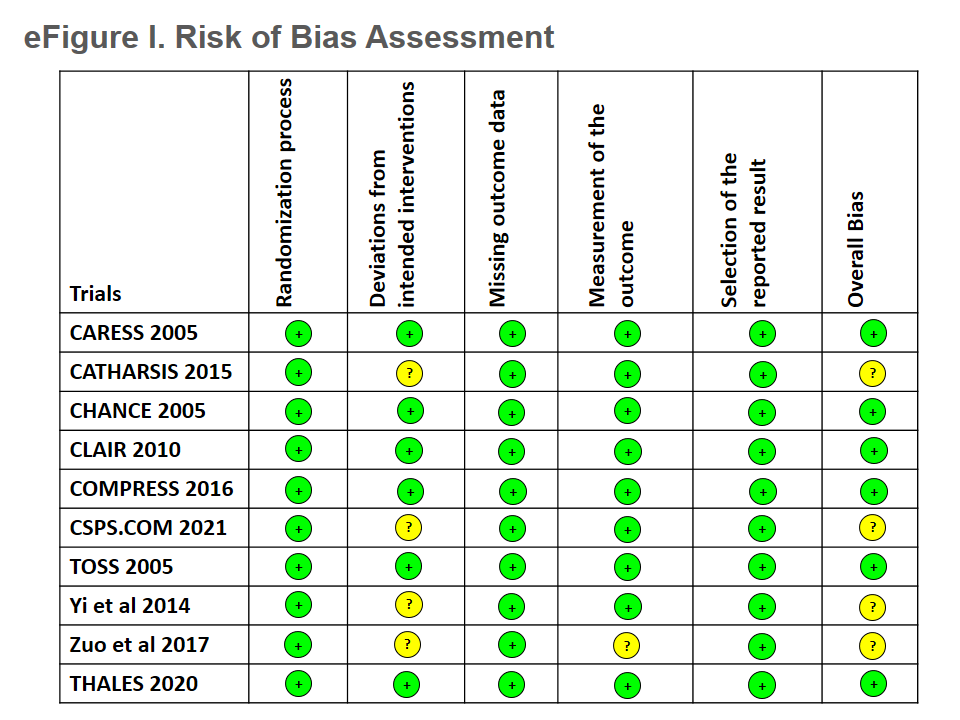


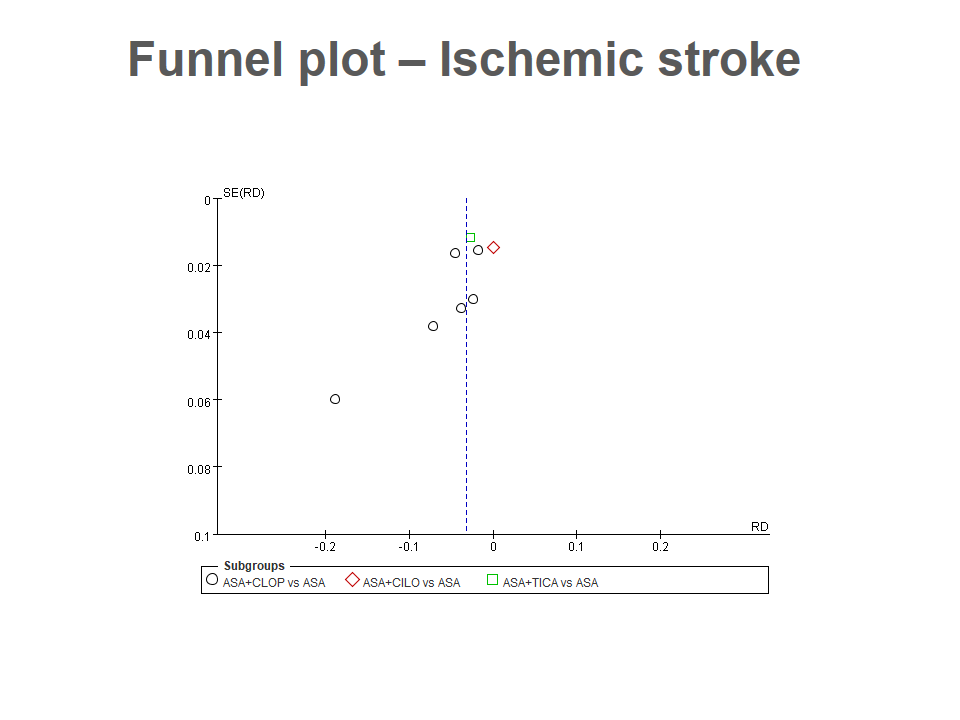
eFigure II. The funnel plots – ischemic stroke. The enrolled studies showed no significant distribution deviation of publication.

eFigure III. The funnel plots – intracranial hemorrhage. The enrolled studies showed no significant distribution deviation of publication.


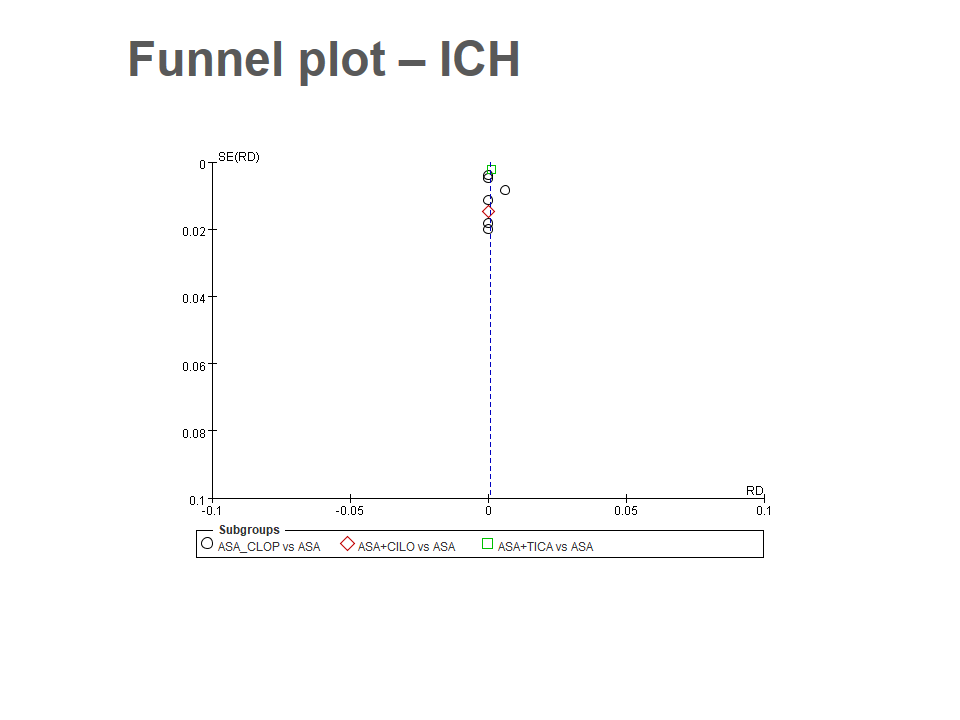


eFigure VI. The funnel plots – major bleeding. The enrolled studies showed no significant distribution deviation of publication.


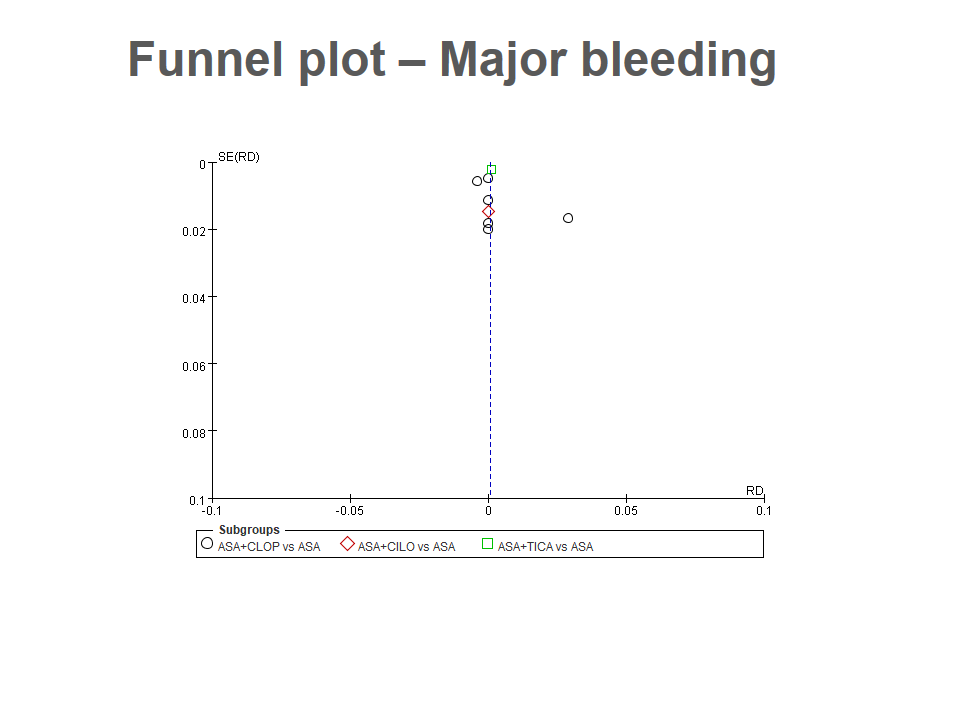

Supplement: Supplementary file 1 [file Data_Sheet_1.docx]
